# 3D-Printed Microfluidic Chip System with Integrated Fluidic Breakers and Phaseguide Fluid Structures for Optimal Passive Mixing

**DOI:** 10.3390/mi17020193

**Published:** 2026-01-31

**Authors:** Christian Neubert, Tim Brauckhoff, Frank T. Hufert, Manfred Weidmann, Gregory Dame

**Affiliations:** 1 Brandenburg Medical School Theodor Fontane, Institute of Microbiology and Virology, 01968 Senftenberg, Germany; christian.neubert@mhb-fontane.de (C.N.); frank.hufert@mhb-fontane.de (F.T.H.); manfred.weidmann@mhb-fontane.de (M.W.); 2 Brandenburg University of Technology Cottbus-Senftenberg, 01968 Senftenberg, Germany; brauctile@disroot.org; 3 Faculty of Health Sciences, Joint Faculty of BTU Cottbus-Senftenberg, MHB Theodor Fontane and University of Potsdam, 14469 Potsdam, Germany; 4 Institute of Animal Hygiene and Veterinary Public Health, Leipzig University, 04103 Leipzig, Germany

**Keywords:** 3D-printed microstructure, microfluidic, passive mixing, Tesla structure, staggered herringbone mixer, split and recombine, phaseguides

## Abstract

3D printing offers great potential for rapid and cost-effective fabrication of microfluidic lab-on-a-chip systems. Through a comparative approach, we implemented staggered herringbone mixer (SHM), Tesla mixer, and split and recombine mixer (SAR), along with a basic unperturbed channel into one chip and performed comparative mixing efficiency experiments. We also introduced a phaseguide-based, T-shaped stop structure at the Y-shaped inlets for bubble-free and parallel filling. The structures were analyzed with two poorly mixable dye solutions at flow rates ranging from 1 µL/min to 200 µL/min. The mixing efficiency was evaluated using optical gray value analysis and compared against diffusion-based mixing. The fluid-aligning phaseguides in the 3D-printed system were shown to work. Among the three different mixing structures tested, SHM exhibited the best mixing efficiency at all tested flow rates. Uniformly designed SHM structures contain a region of poor mixing between the two zones of turbulence. In a non-uniform design, fluid breakers were placed between two SHM units to redirect poorly mixed fluids to the edges, resulting in 100% mixing efficiency across all measured flow rates. These results, especially SHM with fluid breakers, support the development of cost-effective injection-molded lab-on-a-chip systems with improved mixing functionalities at close range instead of simple long-length meandric systems.

## 1. Introduction

Lab-on-a-chip (LOC) systems often leverage microfluidic technologies for applications in biomedical diagnostics and biotechnology processes, such as filling, emptying, and mixing within fluid chambers, which have become increasingly important [1,2]. Due to the small dimensions in simple microfluidic channels, the flow is laminar, resulting in low Reynolds numbers. Because of the absence of turbulence in laminar flow, reagent mixing is based on diffusion, resulting in the need for longer residence times and therefore increased channel lengths [3].

The distance required for complete mixing can be significantly reduced by incorporating mixing structure so that mixing is no longer solely dependent on diffusion. Mixers employed in microfluidic systems can generally be categorized into two types: active and passive mixers [4,5].

Passive micromixers offer a more cost-effective, practical, and easily integrable solution than active micromixers as they do not require an external energy source other than fluid pumping. Mixing is achieved through molecular diffusion and chaotic advection [6]. The interfacial interactions between two laminar fluid streams, and the development of microchannel geometries to enhance mixing performance in short channel lengths, have been studied in great detail [3,4,7,8,9,10,11]. Common problems in passive micromixers include the need for long channels/high residence times for good mixing, challenges with clogging by particulates, poor performance with highly viscous fluids, complex fabrication for intricate designs, and achieving the right balance between high mixing efficiency and low pressure drop. While low-cost, they struggle with slow diffusion, requiring elaborate channel structures (serpentine, obstacles, etc.) to induce chaotic advection, which adds to manufacturing complexity and potential blockages, especially in biomedical uses [10,12,13].

The simplest passive mixers are typically described as T- or Y-junctions [14,15], which simultaneously introduce fluids into a mixing channel. They require high flow velocities to induce turbulence and increasing Reynolds numbers, as, otherwise, the channel would need to be quite long [16]. With a 90° bend immediately following the T- or Y-junction, excellent mixing performance can be achieved at medium Reynolds numbers (Re > 30) [17]. 3D printing of microfluidic systems opens up new possibilities for incorporating passive micromixers with enhanced performance. Several 3D printing technologies are currently available, including FDM (Fused Deposition Modeling), SLA (Stereolithography Apparatus), and inkjet-based 3D printing systems, such as Polyjet and Multijet, which use light-sensitive liquid resins for polymerization [18]. The choice of material and printing method significantly impacts mixing efficiency, as demonstrated by Macdonald et al. [19], who investigated mixing performance at different flow rates (25, 50, and 100 µL/min) using Y-junction microfluidic devices fabricated with FDM, SLA-DLP, and PolyJet systems. The FDM-printed structure exhibited the highest mixing efficiency, attributed to its greater surface roughness, followed by the PolyJet-printed device. The Y-junction in the DLP-SLA chip showed the lowest mixing efficiency, with incomplete mixing observed across all flow rates [19]. Similarly, Zeraatkar et al. [20] investigated meandering/serpentine mixing structures (with 10 loops) designed with Y-shaped inlets using three different 3D printing systems (SLA, FDM, and PolyJet).

### 1.1. Tesla Structure (Transverse Fluid Dispersion)

In 1920, Nikola Tesla patented a structure that he described as a “valvular conduit” that functions as a fluidic pressure reducer and straightener [21,22]. Tesla structures are commonly utilized as valves or pumps in various fluidic applications [23,24]. Hong et al. [25] inverted the original Tesla structure to develop an innovative, in-plane (2D) passive micromixer, integrating the “Coanda effect” as described by Sobey for micro-total analysis systems (µ-TAS) [26]. This modification generates transverse fluid dispersion by reversing the flow direction in Tesla structures, creating a system that induces mixing by splitting the flow into opposing directions, as a flowing fluid adheres to convex surfaces and deviates from its original flow direction by moving along this surface.

As the flow splits and enters opposing directions, successive Tesla modules enhance the mixing efficiency. At the channel’s end, the branched flows collide with the main flow, generating vortices that further facilitate mixing [25]. According to Hong et al., ten Tesla modules achieved nearly 100% mixing efficiency.

### 1.2. SAR Structure (Split and Recombine)

Micromixers employing three-dimensional SAR structures exhibit rapid and homogeneous mixing by enhancing the contact area between the fluids. Similarly to meandric/serpentine and modified Tesla structures, SAR designs demonstrate a reduction in mixing efficiency as flow rates and Reynolds numbers increase [27,28]. SAR mixers can be classified into two main categories, planar (2D) and three-dimensional (3D) configurations, and the latter can be further subdivided into SAR1 (Gray’s configuration) and SAR2 (Chen’s configuration), as described by Habchi et al. [29,30,31].

Notably, Viktorov et al. [6] demonstrated flow-rate-independent mixing efficiency for an H-C mixer, a design featuring an H-shaped module at the center of an upstream chain module. This finding was also corroborated by Enders et al. [32] using 3D-printed approaches. SAR microstructures are complex and often require intricate microfabrication processes [33]. Tran-Minh et al. adopted a simplified 2D planar mixer design, which effectively reduces the diffusion distance between the two fluids [33]. Furthermore, Plevniak et al. explored various SAR designs in 3D printing, showcasing their adaptability and potential for efficient mixing in practical applications [34].

### 1.3. SHM Structure (Staggered Herring Bone Mixer)

The SHM structure disturbs the laminar flow using integrated symmetric (V-shaped) or asymmetric (L-shaped) herringbone structures in the channel, inducing turbulence, which promotes fluid mixing [35,36,37,38,39,40]. In a staggered herringbone mixer, small bar patterns are transversely installed within the mixing channel at a 45° angle relative to the channel walls. After a certain fraction of the total channel length (b = a + c), a 90° bend is introduced, with the shorter section (1/3 of the length) oriented perpendicular to the longer section (2/3 of the length). This asymmetry generates anisotropic flow resistance, which causes the fluids to shift their flow vectors and overlap, resulting in a wave-like rearrangement of the fluids [2,39]. Stroock et al. defined a pattern of six L-shaped grooves as a half cycle, with a laterally reversed L-shaped groove forming a full cycle. After 15 cycles, complete homogeneous mixing of the fluids is achieved [2,36,41].

Kwak et al. [38] examined the conventional “herringbone grooves” (negative variant), convex grooves (positive variant), and two flow direction variants: forward (L-shaped structure facing the flow direction) and reverse (opposite direction).

The positive–forward and positive–reverse SHM configurations were identified as the most efficient, with the conventional negative design requiring four cycles (one half-cycle consists of 10 L-shaped grooves) and exhibiting approximately half of the mixing efficiency of the positive–forward flow variant at a flow rate of 0.36 µL/min [38].

### 1.4. Phaseguides (Concept for Filling and Emptying of 3D-Printed Microfluidic Devices)

Phaseguides are capillary pressure barriers that enable controlled, bubble-free filling and emptying of liquids into microfluidic chambers. These phaseguides consist of thin hydrophilic bars (such as ordyl dryfilm resist) applied perpendicular to the flow direction. When the liquid contacts the phaseguide, a phase meniscus forms along the guide’s length. The abrupt change in capillary pressure at the phaseguide interface ensures that the liquid only proceeds to the next section once the current section is fully filled [42,43,44]. Craig et al. demonstrated the functionality of phaseguides in a 3D-printed setup using a DLP-SLA printer [45].

#### The Study

This study addressed the challenge of developing a simple microfluidic passive mixing structure with few structural elements that would be easy to 3D print. This short-length mixing structure should show the highest mixing efficiency independent from any differences in flow rate or residence time of the fluids in the mixing area. Another challenge is to find a solution to filling microfluidic systems without bubbles.

Unlike conventional meandric/serpentine passive mixing structures, SAR, Tesla, and SHM mixing structures use various approaches to increase the residence time of the liquid by either extending the length of the channels or significantly reducing the flow rates.

− In meander/serpentine channels and modified Tesla and SAR structures, mixing efficiency first fluctuates depending on the flow velocity and increasing Reynolds numbers.
− Depending on flow velocity, asymmetrical SHM structures can form a poorly mixable zone in the middle of the channel, regardless of the number of mixing structures used.
− Highly efficient 3D structures (e.g., helical mixers) can be difficult and costly to fabricate, and complex designs that enhance mixing (e.g., obstacle arrays) can significantly increase pressure drop, requiring more pumping power.
− It is a challenge to achieve bubble-free parallel filling of two fluids in a passive mixing structure.

To test if implementation of passive structures can be performed through 3D printing with biocompatible polymer resin “NextDent Surgical Guide” (NDSG) [46], we implemented 3D-printed SAR, Tesla, SHM structures along with a basic unperturbed channel in one chip and, through comparative mixing efficiency experiments, studied the performance of all three mixing structures.

For precise characterization of the mixing process, two water-soluble dyes with low diffusion coefficients, Congo red and Coomassie blue, were chosen. This allowed for more accurate measurement of mixing efficiencies, isolating the effects of the mixing structures.

Studying the influence of printing artefacts on the mixing efficiency, we showed a possible influence of 3D artefacts on laminar flow in the unperturbed channel without any mixing structures but only marginal effects on the performance of the chosen passive mixing structures.

The 3D-printed Tesla structures demonstrate a loss of mixing efficiencies at higher flow velocities. For the uniformly designed SAR structure, we were not able to demonstrate high mixing efficiencies. The best mixing efficiencies were shown with the uniform, asymmetrical SHM structures, which, however, formed a poorly mixable zone in the middle of the channel.

To circumvent this specific problem, we designed novel geometries with optimized central interrupting arrays to induce better chaotic flow with shorter lengths for low pumping power. Independent of the type of fluidic breaker implemented, we were able to achieve 100% mixing efficiency at flow velocities commonly used in microfluidic systems. For bubble-free and parallel filling, we introduced a phaseguide-based T-shaped stop structure at the Y-shaped inlet of the mixing channel, which clearly enhanced mixing consistency.

## 2. Materials and Methods

### 2.1. Manufacturing 3D-Printed Micromixers

The devices were designed using the computer-aided design (CAD) software Solidworks^®^ 2023 SP2.1 (Dassault Systèmes, Vélizy-Villacoublay, France) and exported to a 3D printing pre-processor/slicer program (NetFabb, Autodesk, San Rafael, CA, USA). The slicing process was performed at a layer thickness of 50 µm using settings specific to tested biocompatible printing material NDSG (NextDent Surgical Guide, Soesterberg, The Netherland) [46]. 3D printing was conducted on a SolFlex SF350 (Way2Production, Vienna, Austria) DLP printer using the PowerVat printing bed. In this study, Gentle was selected as the BuildStyle to print the fine/detailed structures of the micromixers with the highest quality. The 3D-printed devices were first cleaned of residual resin using the cleaning protocol according Behrmann et al. [46]. After the first treatment, the devices were additionally irradiated with a stroboscopic high-intensity light (UV-A and visible waves) at a rate of 10 flashes per second, corresponding to an irradiation time of 5 min with 3000 flashes in the UV-Otoflash G171 apparatus (NK-Optik GmbH, Baierbrunn, Germany). The filter used (type360N2) is transparent to the wavelengths from 360 nm to 700 nm. After the individual preparations, the 3D-printed mixing chips (Figure S1) were stored and protected from light.

### 2.2. Experimental Setup for the Mixing Tests

**Figure 1:**
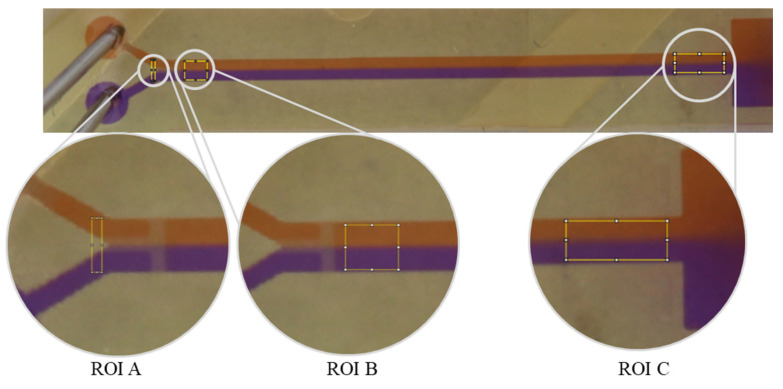
Illustration of the regions of interest (ROI) used for the analysis. (ROI A): Before the fluids meet, the analysis rectangle is drawn to determine the initial state *I*_0_. The analysis rectangle is located before the T-structure and observes the two fluids in the inlet channels. To determine the diffusion of both liquids to avoid any possible mixing effects due to any voxel structures or layers from the printing process in the channel, a second control localized behind the T-stop structure is designated as ROI B. The ROI C for states *I_max_* and *I*_*x*_ is located before the reservoir or after the mixing structure. For orientation, this area is marked with indentations next to the channel. Orange Congo red stained solution, purple Coomassie blue stained solution.

For parallel microfluidic investigation (Figure S1A,B), the Fusion200 pump system was used in combination with an 11-Syringe Infuse/Withdraw Expanding Rack (Chemyx, Stafford, TX, USA) and loaded with 8 1 mL Omnifix-F syringes with appropriate tips (Sterican, B|Braun, Melsungen, Germany). The 3D-printed device was overlayed with a PCR film (Carl Roth, Karlsruhe, Germany) to seal the microfluidic channels. Two options for infusion of the liquids were printed. The first was an inlet pierced with cannula (inner diameter 0.75 mm (Vici-Jour, Schenkon, Switzerland)) showing no leakage regardless of the chosen flow rate (Figure 1 and Figure 4). The second was a round-sided inlet fit to the cannula, as shown in Figure S1C, a design taking the 3D printing procedures into account.

Reproducible images under the same environmental and lighting conditions were essential to obtain comparable results. Measurements were performed in a “photo box” (Styrofoam, Wilmington, DE, USA), as shown in Figure S1B, with high reflectivity, using an EOS 80D SLR camera (Canon, Tokio, Japan) in combination with an EFS 18–55 mm Image Stabilizer (Canon) to ensure high reproducibility of the images with image quality of 24 M (6000 × 4000 pixels).

### 2.3. Measurement of Mixing Efficiencies

Dyes with a low diffusion coefficient and good solubility in water were chosen to reduce unavoidable mixing due to diffusion. Congo red (Sigma, Taufkirchen, Germany) and Coomassie blue (SERVS Electrophoresis GmbH) were used as dyes. Congo red has a diffusion coefficient of 5.67 × 10^−10^ m^2^/s [47], and Coomassie blue has a diffusion coefficient of 1 × 10^−10^ m^2^/s [48]. The concentration used for Congo red (anionic, molecular weight 696,66 g/mol) was 0.2 g/L (0.29 mmol/L), and for Coomassie brilliant blue (anionic, molecular weight 854.04 g/mol) it was 0.14 g/L (0.17 mmol/L). The color solutions were pumped into two separate inlets and introduced in equal volumes into the respective channels. Following flow velocities, 1, 2, 5, 8, 10, 16, 20, 40, 50, 100, and 200 µL/min were set as the experimental parameters and tested fivefold each.

The corresponding Reynolds numbers (Re) in the inlet channel leading to the mixing structures were 0.16, 0.33, 0.666, 1.66, 2.66, 3.33, 6.66, 13.33, 16.66, and 33.33 and calculated according to Equation (1):

(1) $$Re=\frac{\rho \cdot \dot{V}\cdot L_{C}}{w\cdot h\cdot \eta }$$

(2) $$L_{C}=\frac{4\cdot w\cdot h}{2\left(w+h\right)}$$

where ρ denotes the fluid density, $\dot{V}$ is the volumetric flow rate, $L_{C}$ is the characteristic channel dimension, and η is the dynamic viscosity of the fluid. The parameters w and h represent the channel width and height, respectively. The characteristic length $L_{C}$ was determined using Equation (2).

A phaseguide arranged as a T-stop structure aligns both fluids into the main channel, introduced separately via a Y-arranged channel structure [42].

To determine the diffusion of both liquids only and avoid any possible mixing effect due to any voxel structures or layers from the printing process in the channel, a second control localized behind the T-stop structure was designated as ROI B (Figure 1 and Figure 3). For that purpose, the colored fluids were introduced at 100 µL/min, and the pump was stopped when the liquids reached the regions of interest B (see below). Images were taken after specific time points (1, 2, 3, 5, 10, 15, 20, 30, 40, 50, 100, 150, 200, 300, 450, and 600 s).

### 2.4. Determination of Mixing Efficiency Using Image Analysis and Data Processing

Three regions of interest (ROI), marked as A, B, and C (Figure 1), were defined to indicate areas important for analysis and evaluation. Because the microfluidic polymer chip would have its own grayscale values, different areas (ROIs) in the 3D-printed devices were defined for determination of the initial status *I*_0_ (ROI A) and investigation of pure diffusion after the stop structure (ROI B) and after the mixing structure (ROI C) (Figure 1). For the initial state (*I*_0_), the rectangle was placed before the T stop-structure to exclude any mixing that might arise from the meeting of the fluids and the T stop-structure. The unknown mixed state (*I_x_*) and the fully mixed state (*I_max_*) were examined in ROI C (Figure 1) before the reservoir and behind the mixed structure. To determine *I_max_*, both fluids were completely mixed through vortexing (30 s) before they passed through the mixing structure. To compare the mixing efficiency of the different mixing structures, each was analyzed against the fully mixed state (*I_max_*) in the channel.

ROI C was analyzed twice, first to determine the unknown state and second to determine the fully mixed state. Complete mixing was derived through vortexing for 30 s before passing through the mixing structures. Thus, we defined a uniform *I_max_* as the reference point for all mixed structures (Figure 1).

**Figure 2:**
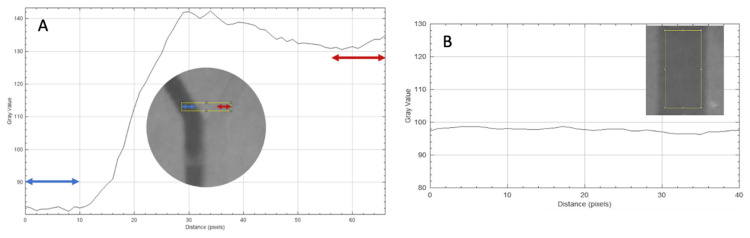
(**A**) **Complete unmixed status.** The analysis rectangle (yellow) (ROI A) before the T-structure. The colored arrows symbolize the different fluids flowing through the inlet channels. A plot showing the grayscale values against the distance in pixels was derived from the analysis rectangle. In the graph, the first and last 10 pixels are marked with arrows. These reflect the fluids present in the inlet. The grayscale values in the marked areas were used for the further calculations. (**B**) **Complete mixed status**. The analysis rectangle (yellow) after the mixing structure (ROI C). The gray values in the ordinate fluctuate between 94 and 96.5.

The analysis was conducted using R programming language version 4.2.1 [49]. The captured images were analyzed using the ImageJ program (version 1.53t). The image was first divided into RGB channels (red, green, blue). The individual color channels were reproduced as 8-bit grayscale JPG images (the original format was retained) [5]. The red color channel was selected for analysis. Due to the selected channel, the red fluid appears light with high grayscale values, and the blue fluid appears dark gray with lower grayscale values. The analysis rectangle was stretched over the region of interest (ROI). The grayscale values were plotted against the horizontal distance in pixels. The grayscale values were averaged row by row/vertically across the analysis rectangle (Figure 2), which offers the advantage of compensating for pixel artifacts in the ROI.

### 2.5. Determination of Initial State I_0_ in ROI A and I_max_ in ROI C

Calculations for mixing efficiency and theoretical considerations: As demonstrated in other publications [50], use of the standard deviation provides a way to estimate the dispersion of grayscale values within the area under consideration. This means that the more varied the grayscale values, the higher the standard deviation, and the lower the mixing of the two fluids.

In the initial state (*I*_0_), the grayscale profiles show two clearly separated plateaus corresponding to the two fluids, resulting in a maximal difference in grayscale values between them. In the fully mixed state (*I_max_*), the grayscale values are nearly identical, and the differences between pixels approach zero.

Before analysis, the first and last ten pixels of the analysis rectangle (ROI A) were taken as representatives for the fluids in their unmixed states. The grayscale values determined along the pixel distance were exported to an R script for further processing.

**Figure 3:**
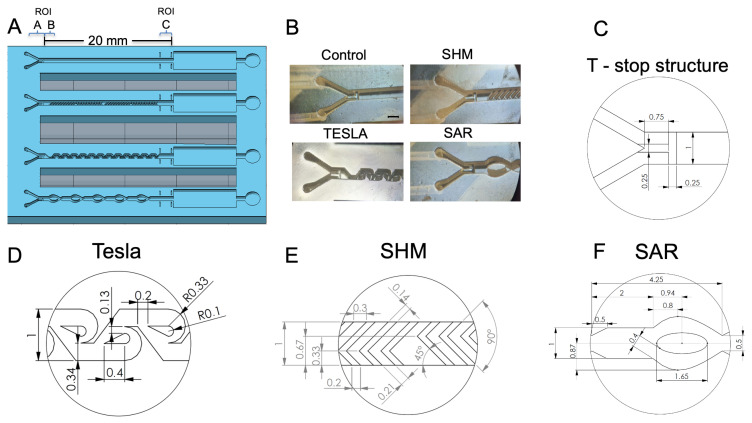
(**A**) Illustration of the 3D-printed test chip, featuring its passive mixing structures, with the regions of interest (ROI) A, B, and C indicated for orientation. (**B**) Microscopic image (35× magnification) showing all of the mixing structures, along with the control channel. (**C**) Schematic representation of the phaseguided T-stop structure. (**D**) Schematic representation of the Tesla structure. (**E**) Schematic representation of the uniform L-shaped-designed staggered herringbone mixer (SHM) structure. (**F**) Schematic representation of the split and recombine (SAR) structure.

To compare the mixing efficiency of the different mixing structures, each was analyzed against the fully mixed state (*I_max_*) in the channel. The standard deviation was determined for three states: the modified initial state (*I*_0_), the unknown state (*I_x_*), and the fully mixed state (*I_max_*). The standard deviation provides a quantitative measure of mixing efficiency, as standard deviation is high in the initial state and low in the fully mixed state and the intermediate state *I_x_* falls accordingly in between.

The mixing efficiency (ME) was calculated as

$$ME=\left(1-\frac{\left(\sigma \left(I_{X}\right)-\sigma \left(I_{max}\right)\right)}{\left(\sigma \left(I_{0}\right)-\sigma \left(I_{max}\right)\right)}\right)\times 100\%$$

$\sigma \left(I_{X}\right)$ = standard deviation of grayscale values in the intermediate state (*I_x_*);

$\sigma \left(I_{0}\right)$ = standard deviation in the unmixed state (*I*_0_);

$\sigma \left(I_{max}\right)$ = standard deviation in the fully mixed state (*I_max_*).

In this formulation, ME = 0% corresponds to the unmixed state and ME = 100% corresponds to the fully mixed reference. This metric reliably captured the transition from unmixed to mixed states across flow rates and structures while compensating for pixel-level artifacts and background noise introduced during the printing process.

## 3. Results

One of the primary objectives of this work was to minimize the dimensions of the mixing structures for optimal chaotic passive mixing while ensuring that the devices demonstrate relatively independent performance across a range of flow rates typically encountered in microfluidic systems. A significant challenge was the integration and realization of specific microfluidic features, such as phaseguides, using a biocompatible resin. These features were designed to be fabricated within the resolution limits of a DLP-based 3D printer, ensuring sufficient shape fidelity in combination with various mixing structures. Several challenges were encountered in achieving prompt and effective filling of the 3D-printed test chip.

### 3.1. 3D-Printed Mixing Device with Chaotic Mixing Features

Three distinct structures (SAR, Tesla, SHM) described as efficient passive mixers were incorporated into a 3D-printed chip, alongside a reference channel devoid of any mixing features [2,25,33]. This experimental setup enables a direct comparison of the different design variations under identical conditions. The simple channel, lacking any mixing structures, serves as a reference both for measurements and to assess the potential influence of voxels [51,52], as well as print-related layer structures (roughness) on mixing efficiency, in comparison to the diffusion control [53].

For practical reasons, an injection port was fabricated on the lateral side of the chip to accommodate the cannulas for parallel fluid injection, as illustrated in Figure S1C. Both fluids were introduced into a central channel via a Y-shaped inlet structure (Figure 3A,B). This design allows for the placement of a PCR film without the need for piercing. In Figure 3A, the regions for determining the measurement areas are labeled as ROI A, which corresponds to the range of unmixed fluids (*I*_0_). The ROI C measuring area is used to analyze the mixing efficiency (*I_x_*) of the various structures and is framed by four printed bars for improved orientation. The square chamber behind serves as a waste chamber, while the adjacent circular chamber is dedicated to venting the system, ensuring that results are not distorted by potential back pressure from compressed air.

Figure 3B shows the 3D-printed realizations as a microscopic image (35× magnification). The top left shows the channel without any mixing structure, the top right shows the SHM structure, the bottom left shows the modified Tesla structure, and the bottom right shows the SAR structure. All structures are visualized with the Y-shaped inlet structure together with the phaseguide T-stop structure. For a more detailed assessment of the various structures, Figure 3D–F provide further information of the individual features and dimensions of the designs created with SOLIDWORKS^TM^.

### 3.2. 3D-Printed T-Stop Structure Based on Phaseguide Technology

**Figure 4:**
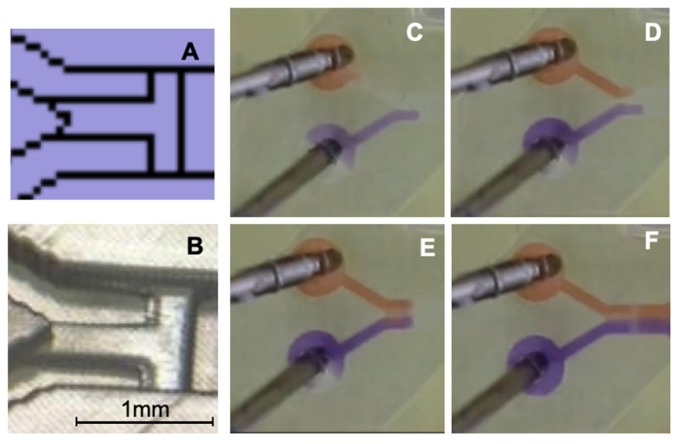
Representation of the phaseguide T-stop structure. (**A**) Section of the printing file. (**B**) Section of the 3D-printed channel; the black bar corresponds to 1 mm. (**C**) Filling with Coomassie blue (lower channel, purple) and stopping at the T-stop structure. (**D**) Filling with Congo red (upper channel, orange) before T-stop structure. (**E**) Both fluids form together a meniscus, and, in (**F**), both fluids overflow the T-stop structure and flow together into the mixing channel.

As an alternative to a valve-switch mechanism described by Melin et al. [54], a stop structure based on phaseguide technology was designed [42]. At the entrance of the mixing channel, a phaseguide structure, functioning as a capillary pressure barrier, is placed perpendicular to the channel. Additionally, another phaseguide, oriented at a 90° angle, divides the channel into two compartments. Figure 4A presents a schematic of the printing file; note that the rectangular features in the printed channels are not caused by any 3D printing dysfunctionalities. Figure 4B shows the corresponding 3D-printed structure. Although both fluids are initiated simultaneously by the pump, due to slight variations in syringe piston movement, the fluids may enter the mixing channel at different times. This design aims to allow independent filling of both chambers while enabling contact between the two fluids without introducing air bubbles, thus enabling the pre-storage of liquids in a straightforward manner.

Because the inflow of both fluids into the 3D-printed device may exhibit behavior similar to passive Y-mixers, the potential impact of unintended mixing can be minimized using the phaseguide T-stop structure. This ensures that both fluids enter the mixing channel in a parallel and controlled manner. Various snapshots from a time-lapse sequence (Figure 4C–F) illustrate the described functionality.

In Figure 4C, the Coomassie blue solution flows first into the channel and is halted at the phaseguide stop structure, which is positioned perpendicular to the flow direction. In Figure 4D, the Congo red solution is introduced via the second inlet, while the Coomassie blue solution remains at its designated stop position. In Figure 4E, both solutions meet at the T-stop structure, forming a meniscus that subsequently overcomes the capillary barrier. Figure 4F demonstrates the synchronized and bubble-free filling of the mixing channel, ensuring controlled fluid entry. This phaseguide structure serves multiple critical functions in regulating the filling process across different mixing channels, particularly at varying flow rates, enabling the assessment of flow-rate-independent mixing structures fabricated via 3D printing. Compared to conventional valve-switching mechanisms described in the literature, this approach offers enhanced robustness and reliability.

### 3.3. Investigation of Mixing Efficiency of Different Passive Mixing Structures

The mixing efficiency of three 3D-printed passive mixing structures (SAR, Tesla, and SHM) and a reference channel without mixing elements (Figure 3A) was evaluated as a function of different flow rates. The investigated flow rates ranged from υ = 1 to 2, 4, 10, 16, 20, 40, 80, 100, and 200 µL/min. Upon passing through the mixing structures, the fluids were directed into the measurement region (ROI C, 2 mm in length), located immediately downstream of the mixing structures (Figure 3A and detailed in Figure 1). Once the fluids completely occupied ROI C, the syringe pump was stopped, and an image of the measurement area was captured. Each experiment was conducted in five independent replicates (*n* = 5). Image analysis and subsequent calculation of the mixing efficiency were carried out as described in the methods by comparing the measured state (*I_x_*) with the completely mixed (*I_max_*) and the completely unmixed state (*I*_0_).

**Figure 5:**
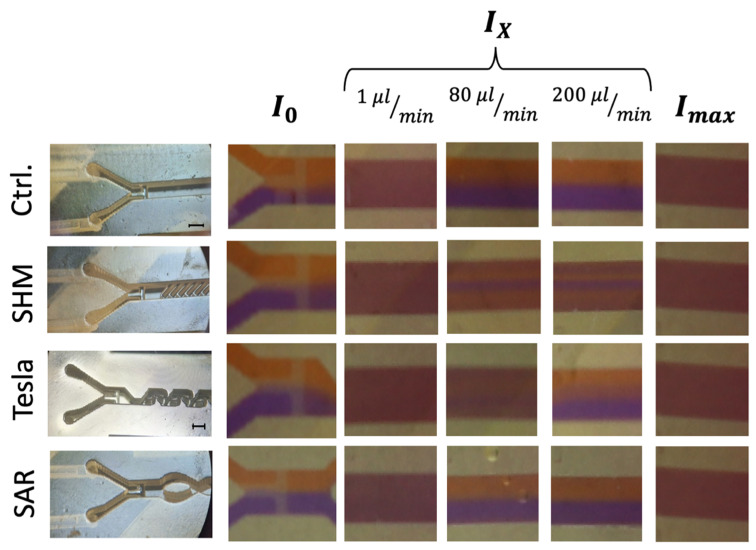
Image-based analysis of ROI C representing the intermediate mixed state (*I_x_*) at different flow rates in comparison to complete (*I_max_*) and unmixed (*I*_0_) states. The black scale bar corresponds to 1 mm. **Left panel:** Microscope images of all mixing structures, categorized into three rows corresponding to *I*_0_ (completely unmixed), *I_x_* (intermediate mixed state), and *I_max_* (completely mixed via vortexing). **Right panel:** Representative still images extracted from video recordings at flow rates of 1, 80, and 200 µL/min, illustrating different mixing states.

A closer examination of the measured grayscale values revealed localized maxima that indicated regions of incomplete mixing. While the applied metric generally captured the transition from unmixed to mixed states across flow rates and structures, certain visual discrepancies between the observed and calculated values remained, particularly at higher flow rates. These inconsistencies were not fully resolved by the grayscale-based analysis method. Figure 5 presents selected frames from video recordings (full videos available in supplementary video section) corresponding to the ROI A, B, and C areas, defined as *I*_0_ (completely unmixed), *I_x_* (intermediate mixed state), and *I_max_* (completely mixed via vortexing), respectively. To illustrate these unresolved differences, especially at flow rates ≥ 80 µL/min, representative snapshots have been included in Figure 5.

**Figure 6:**
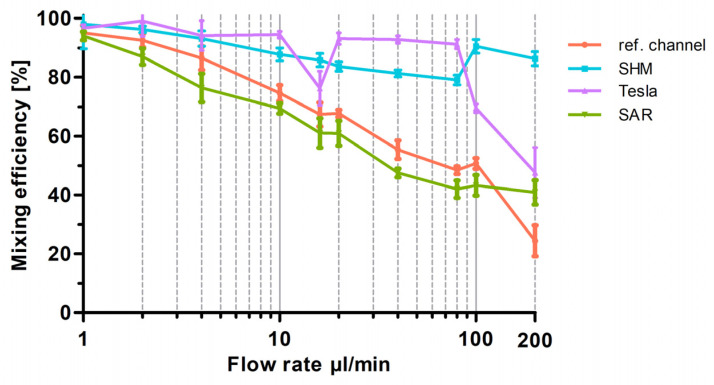
Graphical representation of the optical analysis of mixing efficiency as a function of flow rate for the three different 3D-printed mixing structures (SAR, Tesla, SHM) and the reference channel. Mixing efficiency at flow rates from 1 µL/min up to 200 µL/min are plotted semi-logarithmically. Data are presented as mean ± SD (*n* = 5).

The analysis of *I_x_* largely supports the trends observed in Figure 6, yet it highlights a challenge requiring further investigation. As expected, at the lowest flow rate of 1 µL/min, a diffusion-driven mixing process occurs, resulting in a visual mixing state indistinguishable from the completely vortexed reference, which is also confirmed by the data visualization in Figure 5.

At 80 µL/min, the SAR structure and the reference channel (without mixing structures) exhibit a noticeable separation of the two differently colored fluids, with a diffuse interfacial boundary. However, at 200 µL/min, this diffuse region disappears entirely for both the SAR structure and the reference channel.

In contrast, the Tesla structure demonstrates a relatively homogeneous mixing distribution at 80 µL/min, achieving a mixing efficiency of approximately 90%. However, at 200 µL/min, the mixing performance decreases further to 50%, with the reappearance of fluid separation and a diffuse interfacial boundary, similarly to what was observed in the SAR structure at 80 µL/min (Figure 6).

A more detailed visual analysis of the SHM structure suggests discrepancies with previously published data, particularly at higher flow rates (80 µL/min and 200 µL/min). In both cases, a central unmixed channel of Coomassie-stained fluid remains visibly, indicating that complete mixing is not achieved under these conditions.

A visualized analysis of this chosen SHM mixing structure from 1 µL/min to 2 µL/min shows only a homogeneous distribution. Even at a flow rate of 4 µL/min, a blue region can be observed in the center of the channel in ROI C, which becomes increasingly clearer as the flow rate increases.

As expected, all mixing structures exhibited more effective mixing at lower flow rates up to 20 µL/min than at higher flow rates up to 200 µL/min, following the trend that increasing flow rates reduce diffusion-driven mixing.

To provide a better overview in Figure 6, especially at low flow rates, a semi-logarithmic visualization of the data was chosen for the graph. As shown in Figure 6, at the lowest flow rate of 1 µL/min, all structures show an almost 100% mixing efficiency, representing diffusion-driven mixing of the two fluids. When the flow rate was doubled to 2 µL/min, the SHM and Tesla structures maintained similar efficiencies, whereas the reference channel exhibited a 10% reduction and the SAR structure a 15% reduction, leading to an overall mixing efficiency of 85%. This continuous decline in mixing efficiency with increasing flow rates (1–20 µL/min) is clearly visible in Figure 6. The conducted experiments demonstrate that the SAR structure and the channel without structure exhibit significantly lower mixing efficiencies up to 20 µL/min in comparison to the SHM and Tesla structure. At 10 µL/min, mixing efficiency values were determined as follows: 60% for the SAR structure, 65% for the reference channel, 95% for the Tesla structure, and 90% for the SHM structure. An exception is the measurement of the Tesla structure at a flow rate of around 16 µL/min, where a distinct decrease in mixing efficiency to 75% was noted. To exclude potential artifacts from 3D printing irregularities, the experiment was repeated using a different mixing chip, incorporating additional measurements at 15 and 17 µL/min. This confirmed the observed “negative peak” in mixing efficiency.

With increasing flow rates from 20 to 200 µL/min, the reference channel (without mixing structures) and the SAR structure exhibit a continuous decline in mixing efficiency. Up to 100 µL/min, the SAR structure consistently demonstrates a mixing efficiency approximately 5% lower than that of the reference channel. However, at 200 µL/min, the mixing efficiency of the reference channel drops significantly to 20%, whereas the SAR structure maintains a more stable efficiency of 40%.

The Tesla and SHM mixing structures demonstrate a divergent pattern. At flow rates up to 80 µL/min, the Tesla structure exhibits a high mixing efficiency of around 95%, which undergoes a substantial decline from 100 µL/min to 200 µL/min and drops to 45%. The SHM structure, however, follows an almost opposite trend; while its mixing efficiency is ~85% at 80 µL/min, it increases to approximately 95% as the flow rate rises to 100 and 200 µL/min.

Videos S1–S4 present selected frames from video recordings (see Supplementary Materials). Notably, among the tested 3D-printed mixing structures, the SHM structure stands out as the only design exhibiting a relatively flow-rate-independent mixing efficiency within the tested parameter range.

### 3.4. Investigation of Mixing Efficiency of SHM Structure with Integrated Fluid Breakers

A closer examination of the measured grayscale values revealed that localized maxima appeared as poorly mixed regions. To emphasize these differences, particularly at higher flow rates (≥80 µL/min), representative snapshots are included in Figure 5 column 3. Videos S5–S7 present selected frames from video recordings (see Supplementary Materials).

A key challenge in this study was to modify the mixing channel design with minimal adjustments while achieving optimal flow-rate-independent mixing. Previous studies have observed that a poorly mixed central region was consistently observed at higher flow rates [55], but simply extending the SHM structures only proved to be disadvantageous [56].

**Figure 7:**
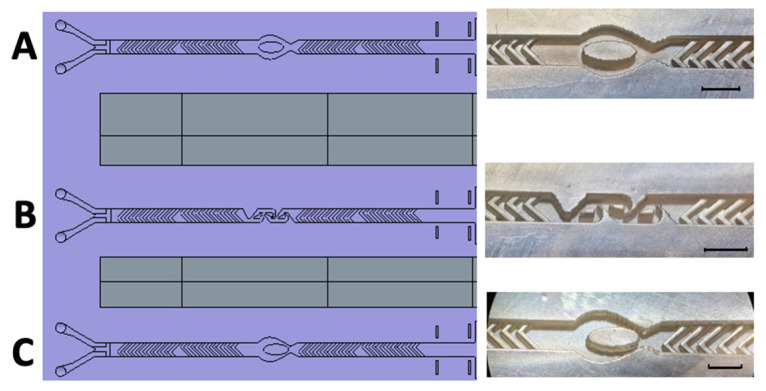
Schematic representation and realized 3D-printed passive mixing structures with integrated fluid breakers. **Left:** Schematic illustration of the newly designed 3D-printed mixing device, incorporating enhanced structural features for optimized passive mixing. **Right:** (**A**) SHM structure combined with a centrally positioned ellipsoid-shaped micropillar. (**B**) SHM structure integrated with three Tesla mixing modules. (**C**) SHM structure featuring an eccentrically positioned ellipsoid-shaped micropillar. The black scale bar corresponds to 1 mm.

Ellipsoidal micropillars have been shown to manipulate fluid flow in simulations [33]. Applying a similar concept, we introduced fluid breakers at strategic positions within the SHM structure to disrupt the poorly mixed central region. We tested the incorporation of SAR structures at various locations (Figure 7A,C) and insertion of a Tesla module as potential fluidic breakers (Figure 7B). The underlying hypothesis was that while SHM structures promote effective mixing at the channel edges, the addition of fluid breakers would force the poorly mixed central region outward, enhancing overall more homogeneous mixing performance.

The second challenge was to implement the new structures without changing the overall channel dimensions. In the initial realization (Figure 3), we followed the approach of Kwak et al. [38] by utilizing two SHM cycles, each containing 10 staggered herringbones per half-cycle, a validated concept described in the literature [38,41]. Stroock et al. [2] demonstrated efficient mixing with just six staggered herringbone grooves per half-cycle, suggesting that a slight reduction—by removing two herringbones per half-cycle—would not significantly impair performance [39].

Thus, in a final design, each SHM module was reduced to eight herringbones per half-cycle, creating sufficient space to integrate the fluid breakers. These fluidic breakers were positioned between the two SHM cycles (Figure 7), ensuring targeted disruption of the poorly mixed central flow region.

### 3.5. Detailed Analysis of Mixing Efficiency Across Uniform and Non-Uniform Mixing Structures

**Figure 8:**
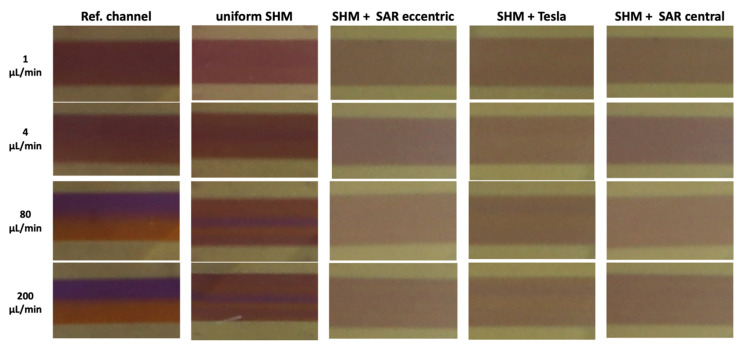
Representative image-based analysis of ROI C extracted from video recordings at flow rates of 1, 4, 80, and 200 µL/min illustrating different homogeneities in mixing state.

An image-based analysis of ROI C extracted from video recordings of the chosen uniform and non-uniform mixing SHM structure and the reference channel at different flow rates from 1 µL/min to 200 µL/min revealed a more or less homologous/heterologous distribution of the liquids (Figure 8).

In the reference channel, a homogeneous distribution of the liquids can be found at a flow rate of 1–2 µL/min. Beginning with 10 µL/min, increasingly stronger splitting of the liquids can be observed, ending in a clear separation at 200 µL/min (Figure 8, column 1). In the uniform SHM approach with asymmetrical structures only, a central unmixed area of the Coomassie fluid is observable beginning at the flow rate of 4 µL/min, which grows more distinct at higher flow rates of 80 and 200 µL/min (Figure 8, column 2).

In the non-uniform SHM structure approach (Figure 8, columns 3–5), much more homogeneous mixing was observed at all flow rates.

**Figure 9:**
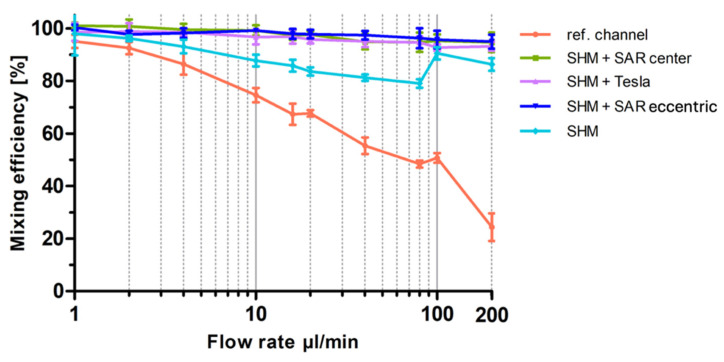
Optical analysis of mixing efficiency as a function of flow rate for newly designed mixing devices with integrated fluidic breakers. The diagram presents the optical analysis of mixing efficiency for the three newly designed mixing devices incorporating fluidic breakers compared to the reference channel without any mixing structures and the SHM structure without fluidic breakers. Mixing efficiency at flow rates from 1 µL/min up to 200 µL/min are plotted semi-logarithmically. Data are presented as mean ± SD (*n* = 5).

Figure 9 presents the measured mixing efficiencies of various non-uniform mixing modules in comparison to the uniform SHM structure, and the reference channel is shown. The results demonstrate that the mixing of the two highly water-soluble but poorly mixable dyes is achieved effectively across all tested structures and flow rates in comparison to the SHM mixing structure without a fluid breaker. Also, the slight decrease in mixing efficiency observed in the symmetric SHM structure between 2 µL/min and 80 µL/min is no longer observed in the modified designs.

## 4. Discussion

Additive manufacturing and 3D printing provide a versatile platform for iterative design modifications and have been described in the literature as suitable tools for investigating microfluidic mixing structures. Two primary approaches have been described. 3D-printed materials can be used as negative templates for PDMS-based positive mixing structures, or they can be directly printed and utilized for experiments. Various printing techniques are available, such as inkjet-based and SLA-based methods, and, in particular, DLP-based systems, all showing advantages in directly fabricating mixing structures while minimizing structural artifacts introduced by the 3D printing process itself [18,19,20,32,46,53].

In preliminary work, monolithically printed chips were developed [46,57]. However, this approach was not pursued here, as the intricate mixing structures within the channels, combined with the washing protocols used here, would not guarantee complete removal of resin residues, potentially contributing to artifacts in the investigation of the mixing structures.

### 4.1. Reference Channel Without Mixing Structure

Macdonald et al. [19] reported that when comparing a 3D-printed Y-junction microfluidic device (FDM (channel width: 0.3 mm), PolyJet (channel width: 0.2 mm), and DLP-SLA (channel width: 0.15 mm), DLP-SLA chips exhibited the lowest level of mixing, with complete mixing not being achieved at any of the examined flow rates (only reaching full mixing at 1 µL/min). Our DLP-printed reference channel with a width of 1 mm exhibited a mixing efficiency of 45 ± 3% at a flow rate of 25 µL/min over a similar length (20 mm; Figure 3A compared to 25 mm in Macdonald et al. These findings suggest that low surface roughness and high design reproducibility in 3D-printed microfluidic devices provide flow profiles. Consequently, devices requiring laminar flow and minimal mixing can be fabricated using DLP 3D printing systems.

In our DLP printing trials, only the resin NextDent Orange resulted in satisfactory precisely realized structures, whereas resins SolFlex Surgical Guide, NextDent Surgical Guide, and NextDent Ortho Clear did not allow for proper implementation.

At higher magnification (shown in Figure S2B), two distinct structural features become apparent in the reference channel. One feature observed in the X–Y direction appears to be a remnant of the pixel-based resolution of the DLP printer, forming aligned pillars in a 3D format. These physically self-aligning units have been described as “voxels” (3D pixels) [51,52]. The observed voxel structures, originating from stacked pixels, analogous to a compound eye, lead to maximal light scattering within the structure and improved roughness of the surface.

The second structural feature in the Z orientation results from the layer-by-layer printing process. This dependency arises from the mechanical interaction between the printing platform and the spindle, as well as the necessary reconfiguration of the platform during resin handling after each fixation step, which leads to potential delamination artifacts, as described by Subirada et al. and illustrated in their study (Figure S2A). They attributed sidewall roughness caused by the DLP-based SLA printing process to laminar displacement of unpolymerized resin, which is washed away during the process so that polymerized resin is left [53]. All of these mentioned structural features, often referred to as “roughness,” may influence mixing efficiency.

### 4.2. Analysis of Mixing Efficiencies Compared to Diffusion Control as a Function of Residence Time

As demonstrated, for example, by Macdonald et al. and Zeraatkar et al., various 3D printer systems introduce varying degrees of surface roughness, which can influence the mixing of two fluids [19,20].

To assess any potential impact of surface structures introduced by DLP printing on mixing efficiency, “pure” diffusion was analyzed in the absence of active flow in the reference channel at ROI B directly after the fluids arrived behind the T-stop structure (Figure S2B).

Because the data from the flow test (ROI C) and the non-flow test (ROI B) of the reference channel do not fully correspond, it can be concluded that voxels, such as the lamellar structures in the sidewalls, possibly have an influence on the mixing process, although a clean channel without voxels with smooth walls was not available for comparison.

**Figure 10:**
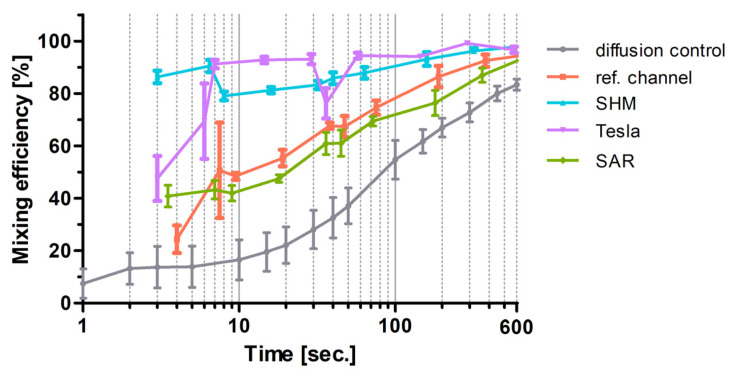
Semi-logarithmic plot of channel residence time (s) against mixing efficiencies (%). The time refers to the residence time of the fluids in the channels between the T-stop structure and the ROI C. The mean value ± standard deviation is shown (n = 5). Lower flow rates (1–20 µL/min) show higher residence time in the mixing structures and higher flow rates of 20–200 µL/min and vice versa. Diffusion control is the measurement in ROI B of the pure diffusion of the two fluids without any passive mixing.

For the sake of comparison, the flow data of the experiments plotted in Figure 6 for SAR, Tesla, SHM, and the reference channel measured at ROI C were adapted and plotted against the residence time into Figure 10 semi-logarithmically. Diffusion-driven mixing efficiency indeed steadily rises and reached 100% at 4000 s (out with the *x*-axis section shown).

The flow data of the SAR channel hardly differ from the reference channel data measured at ROI B, indicating mixing dominated by diffusion in these non-actuated experiments. In contrast, times between fluids until high mixing rates achieved in Tesla and SHM structures are shorter by approximately two orders of magnitude as the flow rate increases, which indicates a dominance of turbulence over diffusion in these systems.

### 4.3. SAR Structure

The model structure presented by Tran-Minh et al. [33] achieved mixing efficiencies greater than 80% at low Reynolds numbers (Re ≤ 1). In our study, the SAR structure resulted in lower mixing efficiencies, even below those of the reference channel. Two distinct behaviors emerged. At higher residence times (100 s or more), the mixing efficiency of both the reference channel and the SAR structure, as well as the diffusion control, cannot be distinguished. At shorter residence times, the SAR structure performs slightly better, similarly to the reference channel. It is important to note that the SAR structure was originally designed for mixing particles in a non-Newtonian fluid (blood), which was directed by two other fluids in a trifluidic concept toward a centrally narrowed stream that passed through an ellipsoidal micropillar [33].

In contrast, our setup uses only two fluids, which may account for the observed decline in performance.

### 4.4. Tesla Structure

The 3D-printed Tesla structure, based on the modified Tesla concept of Hong et al., demonstrated excellent mixing efficiencies, exceeding 90%. When comparing the residence times directly with the diffusion control, as illustrated in Figure 10, the Tesla structure consistently influenced the mixing of the two fluids, regardless of the flow rate. However, two notable exceptions showed a significant reduction in mixing efficiencies. At a flow rate of 16 µL/min, a negative peak in efficiency is observed, and at flow rates exceeding 80 µL/min, the efficiencies drop from approximately 95% to 50% at 200 µL/min (Figure 6).

A combination of two potential explanations for the reduction in mixing efficiency at 16 µL/min in the Tesla structure can be proposed. First, the mixing efficiency drop might be due to an imbalance in flow rate and residence time at low flow rates. Second, due to potential 3D printing artifacts, certain features of the structure might exhibit reduced functionalities.

Hossain et al. [58,59], who specifically investigated Tesla structures, report a similar mixing efficiency drop. This mixing efficiency drop is also evident in other work on SAR and Tesla structures [6,7,25,60]. All of these studies describe the following pattern: initially, higher mixing efficiency at very low flow rates or low Reynolds numbers, followed by a decrease in efficiency as flow rates increase, and then an increase in mixing efficiency at specific flow rates and Reynolds numbers, depending on the investigated system. The explanation for this behavior is that low Reynolds numbers result in a higher residence time, allowing for sufficient time for mixing via diffusion. As Reynolds numbers increase, residence time decreases, causing a drastic drop in mixing efficiency. However, if Reynolds numbers reach high enough values, turbulent flows enhance mixing, which leads to a rise in efficiency. The specific characteristics and corresponding Reynolds numbers can vary between systems and indeed may be subject to the structural features of the systems [7]. Only Enders et al. did not observe the drop in mixing efficiency; having begun their measurements at flow rates ≥ 50 µL/min, they did not observe this effect of low flow rates [32].

**Figure 11:**
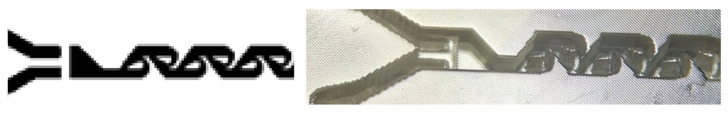
**(Left**) an enlarged section of the print file of the Tesla structure. (**Right**) an enlarged section of the 3D-printed realization.

Numerous analyses of Tesla structures were conducted, working with positive imprints of PDMS from SU8 materials. In our 3D-printed structures, the mixing efficiency decreases significantly at higher flow rates (from 80 µL/min, Figure 6) and low residence times (less than 10 s, Figure 10). Whereas Enders et al. documented a consistent enhancement in mixing efficiency with increasing flow rate, we observed a decline of the mixing efficiency exhibited from 100 μL/min. In our experiments, we used the resin NextDent Orange, which was not tested in the Multijet 3D printing system trials by Enders et al. [32]. Thus, the possibility of a resin-specific influence cannot be excluded. Indeed, the flow-rate-dependent performance of the Tesla structure might be negatively influenced by voxelization artifacts, as well as the lamellar structures in the sidewalls resulting from the 3D printing DLP process and potential resin-related issues (artifacts). Detailed analysis of the print file reveals possible weaknesses in the printing process, including noticeable pixelation, particularly in the wing structure, which were replicated in the 3D-printed device (Figure 11).

Variations in the wing feature design can significantly impact the overall performance of the mixing structure [60,61]. Therefore, it can be hypothesized that the angular features of the wing structures may influence the performance of the Tesla structure, particularly at higher flow rates. It appears that the “Coanda effect” no longer occurs in this 3D-printed version at a certain flow rate.

### 4.5. SHM Structures with Uniform Geometry Conception

Based on prior experience with phaseguide technology, printing constraints, and data derived from Kwak et al. [38], a positive–forward herringbone pattern, referred to as convex grooves, was used instead of SHM grooves employed by others [36,38,40,41]. To account for the resolution capacity of the 3D printing process, the dimensions were enlarged in comparison to the literature but retained similar geometric proportions. A more accurate description of the structure would be the uniform unit cells consisting of asymmetrical L-shaped phaseguide-like rail patterns, with 10 ribbons followed by a further reversed group of 10 angled ribbons in series, as described by [39].

Among all of the structures investigated, the uniform SHM structure demonstrated the highest mixing efficiencies across all tested flow rates. This finding aligns with previous studies utilizing a uniform SHM unit cell concept [36,38,40,41]. Two cycles of a uniform unit cell, arranged in a series of positive–forward convex grooves, were sufficient to achieve complete mixing at a flow rate of 0.36 µL/min, as reported by Kwak et al. [38].

In our 3D-printed chip, with the same number of cycles and at lower flow rates of 1 and 2 µL/min, we observe a homogenous distribution of the dye solutions at ROI C and corresponding grayscale evaluations. At flow rates beyond ≥4 μL/min, a central column of poorly mixed fluid is observable (Figure 5 and Figure 8).

Toth et al. [55] show similar mixing results in a top view of asymmetric uniform SHM structure grooves and describe this as “a multilayer streamline structure that develops due to the rotational effects and the developing transverse advection due to the special microchannel geometry.” In a promising approach, Hadjigeorgiou et al. [39] simulated the asymmetric uniform geometry with six asymmetric units similar to those used by Stroock et al. [2] and, by involving symmetric V-shaped units, demonstrated faster and more homogeneous mixing [39].

### 4.6. Non-Uniform Geometry SHM Structure Conception with Fluidic Breakers

A key challenge in this study was to modify the mixing channel design with minimal adjustments while achieving optimal flow-rate-independent mixing. In previous studies utilizing a µ-TAS with six SHM cycles, a poorly mixed central region was consistently observed at higher flow rates [56]. Simply extending the SHM structure proved disadvantageous, likely due to the formation of dead volumes caused by the numerous herringbone grooves, significantly impacting the Residence Time Distribution (RTD) [62].

To address this issue, this study drew inspiration from the work of Tran-Minh et al. [33], and, applying a similar concept, we introduced fluid breakers at strategic positions within the SHM structure to disrupt the poorly mixed central region. Specifically, SAR structures were incorporated at various locations (Figure 7A,C), while a Tesla module was also tested as a potential fluidic breaker (Figure 7B). The underlying hypothesis was that while SHM structures promote effective mixing at the channel edges, the addition of fluid breakers would force the poorly mixed central region outward, enhancing overall more homogeneous mixing performance. Indeed, we achieved optimal mixing with configurations of centrally positioned pillars between two SHM modules (Figure 9).

## 5. Conclusions

This study highlights the potential of 3D printing technologies, specifically SLA- and DLP-based systems, in the design and optimization of microfluidic mixing structures. Among the various printing methods, DLP-based systems, particularly those from Way2Production (W2P), demonstrated superior performance in the fabrication of microfluidic mixing structures, surpassing SLA-based printers like the Form 3 in terms of resolution and structural integrity.

However, challenges related to resin residue and printing artifacts, such as the formation of voxel-like structures and layer-specific roughness, were identified. These factors could introduce artifacts, affecting the accuracy of mixing efficiency measurements, particularly when working with intricate designs. Despite these challenges, the Tesla and SHM structures showed considerable promise, with the Tesla structure achieving impressive mixing efficiencies of over 90% at lower flow rates, albeit with a noticeable decline in efficiency at higher flow rates.

Mixing efficiency was evaluated using image analysis techniques and assessing the standard deviation of grayscale values in conjunction with residence time analysis. This approach yielded detailed insights into the fluid dynamics within the tested devices and proved robust for quantifying mixing efficiencies, capturing both diffusive and advective contributions while minimizing potential artifacts introduced during the 3D printing process.

The findings suggest that the performance of 3D-printed mixers is influenced by several factors, including channel resolution, material properties, and the specific design of the mixing structure. Notably, the SHM structure, with its asymmetric, uniform design, exhibited the highest mixing efficiency across all tested flow rates, further underscoring the importance of geometry in microfluidic mixing. Additionally, this study supports the notion that the effectiveness of these mixing structures varies with flow rates, with lower flow rates leading to higher mixing efficiencies due to increased residence times.

Testing central breaker structures to disrupt a central volume column non-mixing zone observed in the SHM structure successfully yielded highly efficient mixing independent of the breaker structure chosen. This new insight should help to improve the design of shorter passive mixing structures in the future. Introducing a phaseguide at the Y-inlet provides a solution for bubble-free filling of fluids at the inlet.

In conclusion, this study reinforces the significant potential of 3D printing for microfluidic applications in general and passive mixing structures in particular while highlighting the need for careful consideration of printing artifacts and material properties. Future work should focus on optimizing printing processes to minimize these artifacts and further explore the application of SHM and Tesla structures in a wide range of applications of LOC microfluidic technologies in the context of biomedical and chemical analysis, e.g., in single cell analysis or bacterial diagnostics. In particular, testing non-Newtonian fluids, such as blood, for (single) analysis would be very interesting.

## Data Availability

The original contributions presented in the study are included in the article. Further inquiries can be directed to the corresponding author.

## Supplementary Materials

The following supporting information can be downloaded at https://www.mdpi.com/article/10.3390/mi17020193/s1, Figure S1: Mounting of the 3D-printed chip and measurement setup; Figure S2: “Roughness” structure of the 3D-printed material; Video S1: Reference channel at 20 µL/min; Video S2: Uniform SHM structure at 20 µL/min; Video S3: Uniform Tesla structure at 20 µL/min; Video S4: Uniform SAR structure with central pillar at 20 µL/min; Video S5: SHM structure with integrated SAR (central pillar) at 20 µL/min; Video S6: SHM structure with integrated SAR (eccentric pillar) at 20 µL/min; Video S7: SHM structure with integrated Tesla module at 20 µL/min.

## Abbreviations

The following abbreviations are used in this manuscript:

SHM	Staggered herringbone mixer
SAR	Split and recombine
LOC	Lab-on-a-chip
PDMS	Polydimethylsiloxan
DLP	Digital Light Processing (printer)
SLA	Stereolithography Apparatus
FDM	Fused Deposition Modeling
ME	Mixing efficiency
*Re*	Reynolds number
*P*	Pressure
SU8	Epoxy-based negative photoresist
ROI	Region of interest
µ-TAS	Micro-total analysis system
RTD	Residence Time Distribution
x, y, z	Cartesian coordinates
*I*_0_	Unmixed fluids
*I_max_*	Fully mixed fluids
*I_x_*	Intermediate mixing status
*RGB*	Red, green, blue
JPG	Joint Photographic (Experts) Group
NDSG	NextDent Surgical Guide
RPA	Recombinase Polymerase Amplification
sd	Standard deviation
	Greek symbols
υ	Velocity
*μ*	Fluid dynamic viscosity (kg·m^−1^·s^−1^)
*ρ*	Fluid density (kg/m^3^)
*σ*	Standard deviation
*σ_E_*	Mixing index
