# Supplementary material for: 3D-Printed Microfluidic Chip System with Integrated Fluidic Breakers and Phaseguide Fluid Structures for Optimal Passive Mixing
Source: Micromachines (Basel). 2026 Jan 31;17(2):193. doi: 10.3390/mi17020193 (PMC12943354; doi:10.3390/mi17020193)
Supplement: Supplementary file 1 [file micromachines-17-00193-s001.zip › Supplement Figure S2.pdf]

Supplement

3D-Printed Microfluidic Chip System with Integrated Fluidic Breakers and Phaseguide Fluid Structures for Optimal Passive Mixing

Christian Neubert [1], Tim Brauckhoff [2], Frank T. Hufert [1,2,3], Manfred Weidmann [1,4] and Gregory Dame [1,3,*]

[1] Brandenburg Medical School Theodor Fontane, Institute of Microbiology and Virology, 01968 Senftenberg, Germany; christian.neubert@mhb-fontane.de (C.N.); frank.hufert@mhb-fontane.de (F.T.H.); manfred.weidmann@mhb-fontane.de (M.W.)
[2] Brandenburg University of Technology Cottbus-Senftenberg, 01968 Senftenberg, Germany; brauctile@disroot.org
[3] Faculty of Health Sciences, Joint Faculty of BTU Cottbus-Senftenberg, MHB Theodor Fontane and University of Potsdam, 14469 Potsdam, Germany
[4] Institute of Animal Hygiene and Veterinary Public Health, Leipzig University, 04103 Leipzig, Germany
* Correspondence: gregory.dame@mhb-fontane.de

“Roughness” structure of the 3D-printed material

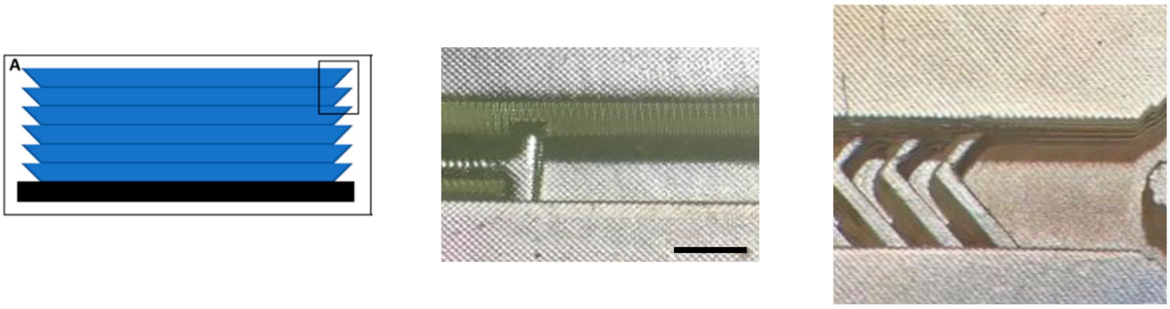

(A) (B) (C)

Figure S2. (A) Example of a 3D printed part and its possible sidewall roughness (altered from Subirada et al. 2020) (B) snapshot of microscopical picture (200X) of the channel without mixing structure behind the T-stop structure. On the top surface the planar voxel structure and the sidewall with vertical orientated rough structures. (C) snapshot of microscopical picture from the sidewall
