# Supplementary material for: 3D-Printed Microfluidic Chip System with Integrated Fluidic Breakers and Phaseguide Fluid Structures for Optimal Passive Mixing
Source: Micromachines (Basel). 2026 Jan 31;17(2):193. doi: 10.3390/mi17020193 (PMC12943354; doi:10.3390/mi17020193)
Supplement: Supplementary file 1 [file micromachines-17-00193-s001.zip › Supplement_190325 Figure S1.pdf]

Supplement

3D-Printed Microfluidic Chip System with Integrated Fluidic Breakers and Phaseguide Fluid Structures for Optimal Passive Mixing

Christian Neubert [1], Tim Brauckhoff [2], Frank T. Hufert [1,2,3], Manfred Weidmann [1,4] and Gregory Dame [1,3,*]

[1] Brandenburg Medical School Theodor Fontane, Institute of Microbiology and Virology, 01968 Senftenberg, Germany; christian.neubert@mhb-fontane.de (C.N.); frank.hufert@mhb-fontane.de (F.T.H.); manfred.weidmann@mhb-fontane.de (M.W.)
[2] Brandenburg University of Technology Cottbus-Senftenberg, 01968 Senftenberg, Germany; brauctile@disroot.org
[3] Faculty of Health Sciences, Joint Faculty of BTU Cottbus-Senftenberg, MHB Theodor Fontane and University of Potsdam, 14469 Potsdam, Germany
[4] Institute of Animal Hygiene and Veterinary Public Health, Leipzig University, 04103 Leipzig, Germany
* Correspondence: gregory.dame@mhb-fontane.de

Mounting of the 3D-printed Chip and Measurement setup

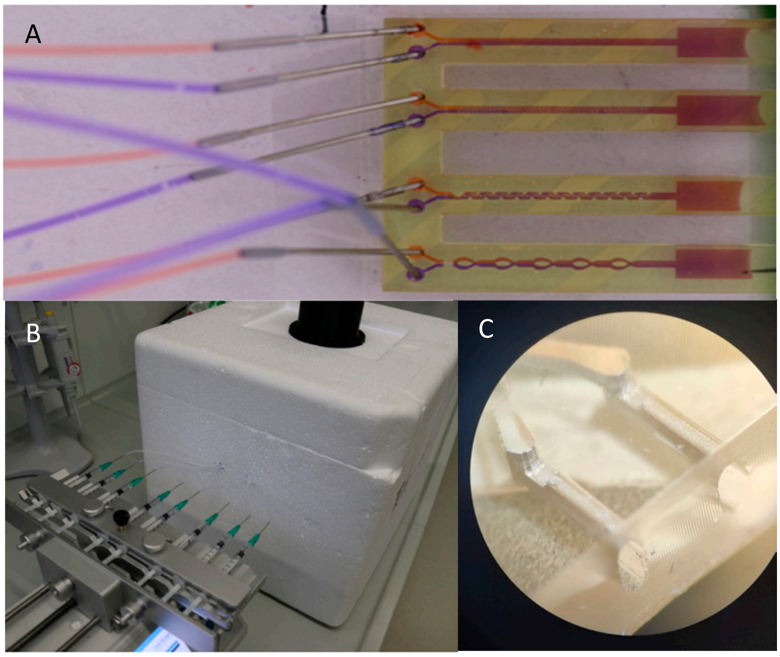

Figure S1: Mounting of the 3D-printed Chip **A)** on top view **B)** Styrofoam photo box, pump inlet, 3D-printed camera holder **C)** An enlarged section of the lateral cannula input into the chip
