# Supplementary material for: 3D-Printed Microfluidic Chip System with Integrated Fluidic Breakers and Phaseguide Fluid Structures for Optimal Passive Mixing
Source: Micromachines (Basel). 2026 Jan 31;17(2):193. doi: 10.3390/mi17020193 (PMC12943354; doi:10.3390/mi17020193)
Supplement: Supplementary file 1 [file micromachines-17-00193-s001.zip › supplement_micromachines_Version Video.pdf]

Video S1: Reference channel at 20 µL/min, showing laminar flow without mixing structures.

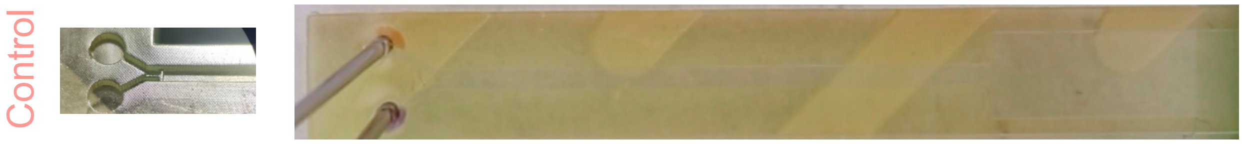

Video S2: Uniform SHM structure at 20 µL/min, demonstrating transverse advection at channel edges.

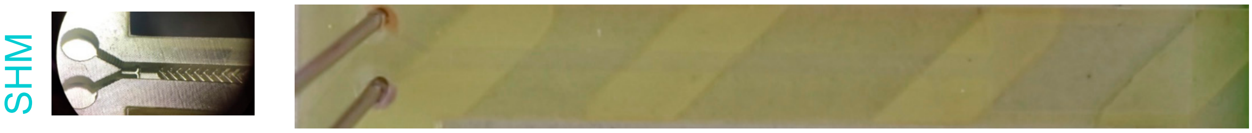

Video S3: Uniform TESLA structure at 20 µL/min, showing fluid folding and redirection.

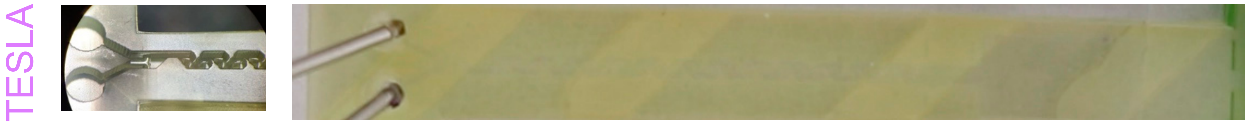

Video S4: Uniform SAR structure with central pillar at 20 µL/min, showing partial fluid splitting.

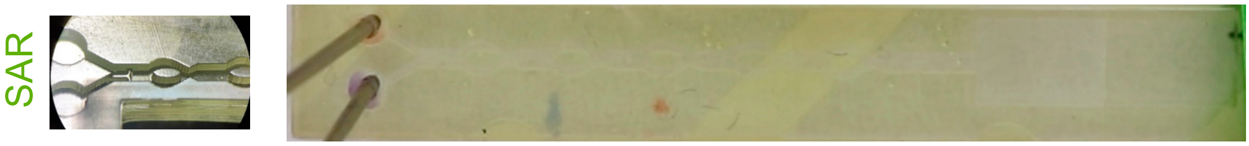

Video S5: SHM structure with integrated SAR (central pillar) at 20 µL/min, reducing central unmixed regions.

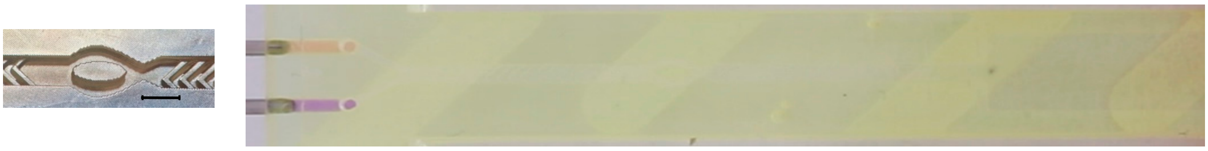

Video S6: SHM structure with integrated SAR (eccentric pillar) at 20 µL/min, altering flow deflection.

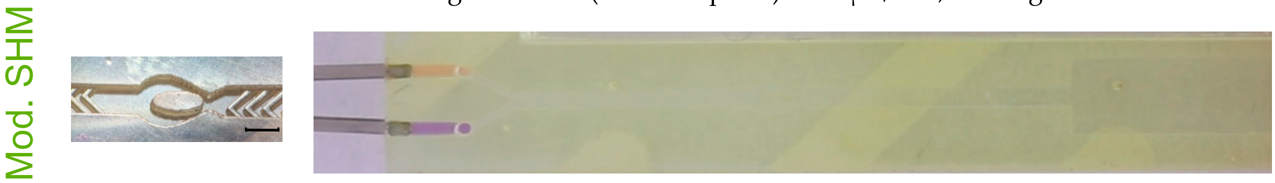

Video S7: SHM structure with integrated TESLA module at 20 µL/min, combining herringbone and TESLA effects for improved mixing.

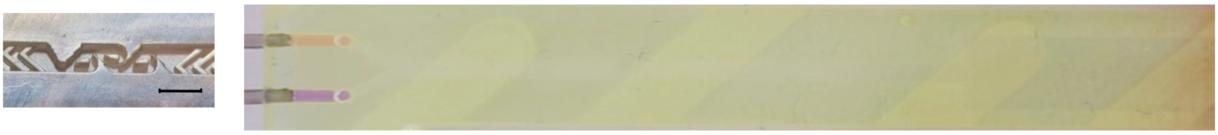

Videos S1 – S7: selected video examples.

First four videos represents inlet with pierced PCR foil with cannula. In the last three videos the liquids are filled from the side inlet (Figure S1b). Regardless which filling method was chosen the various functionalities such as T-stop phaseguide structure or

following mixing structures show no differences. In particular, the functionality of the T-stop phaseguide structure can be observed here in detail.
