# Supplementary figures and images for: 3D-Printed Microfluidic Chip System with Integrated Fluidic Breakers and Phaseguide Fluid Structures for Optimal Passive Mixing
Source: Micromachines (Basel). 2026 Jan 31;17(2):193. doi: 10.3390/mi17020193 (PMC12943354; doi:10.3390/mi17020193)

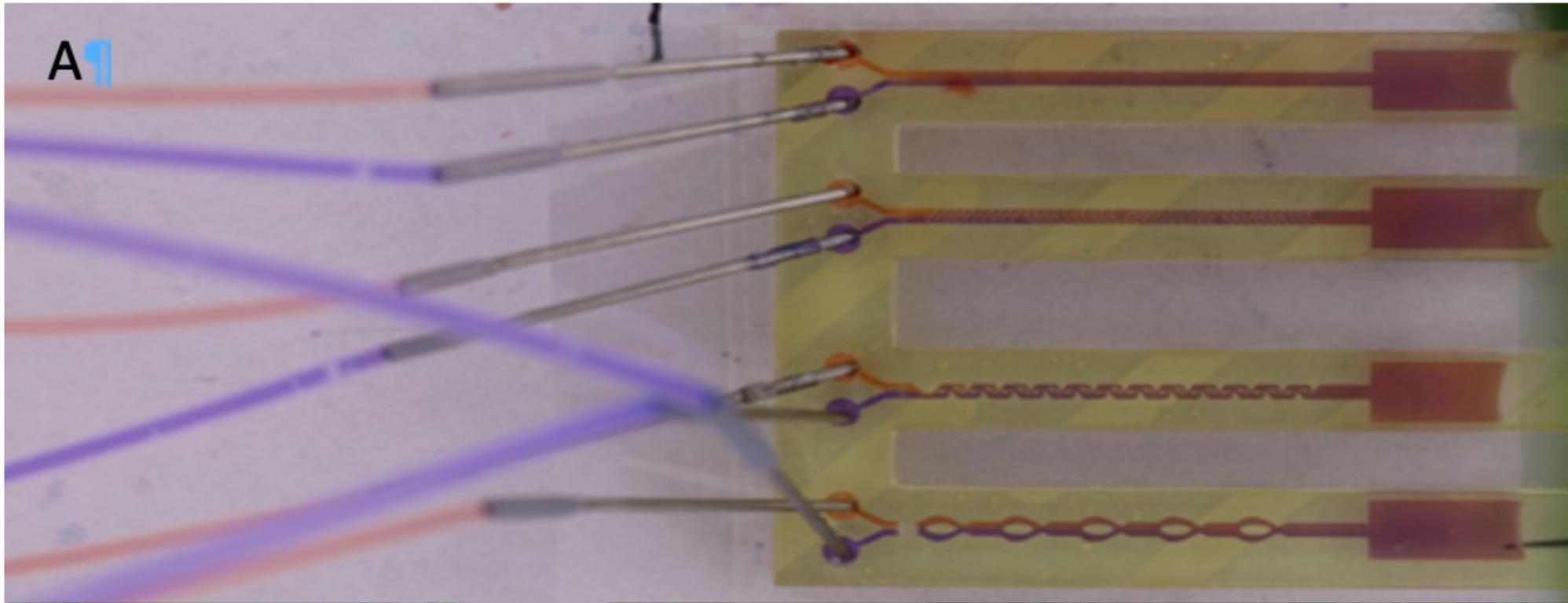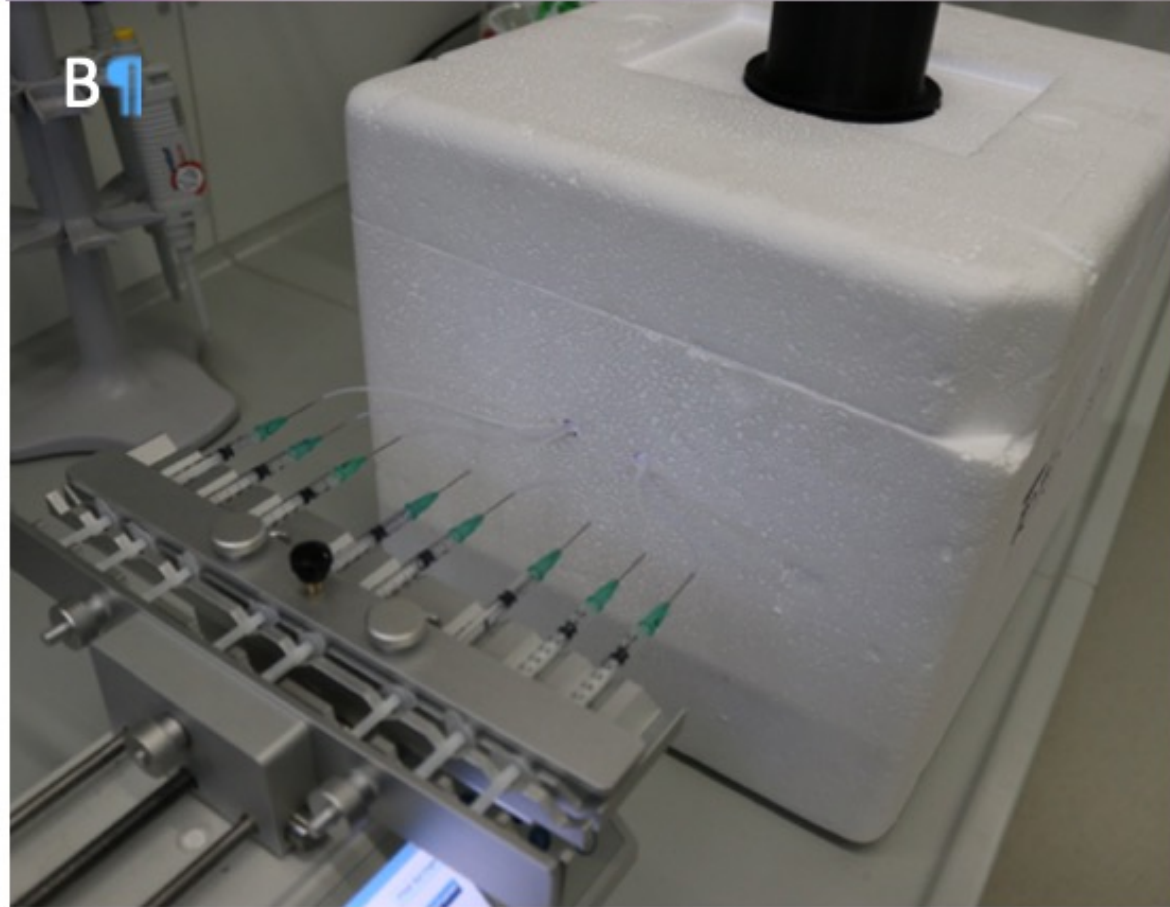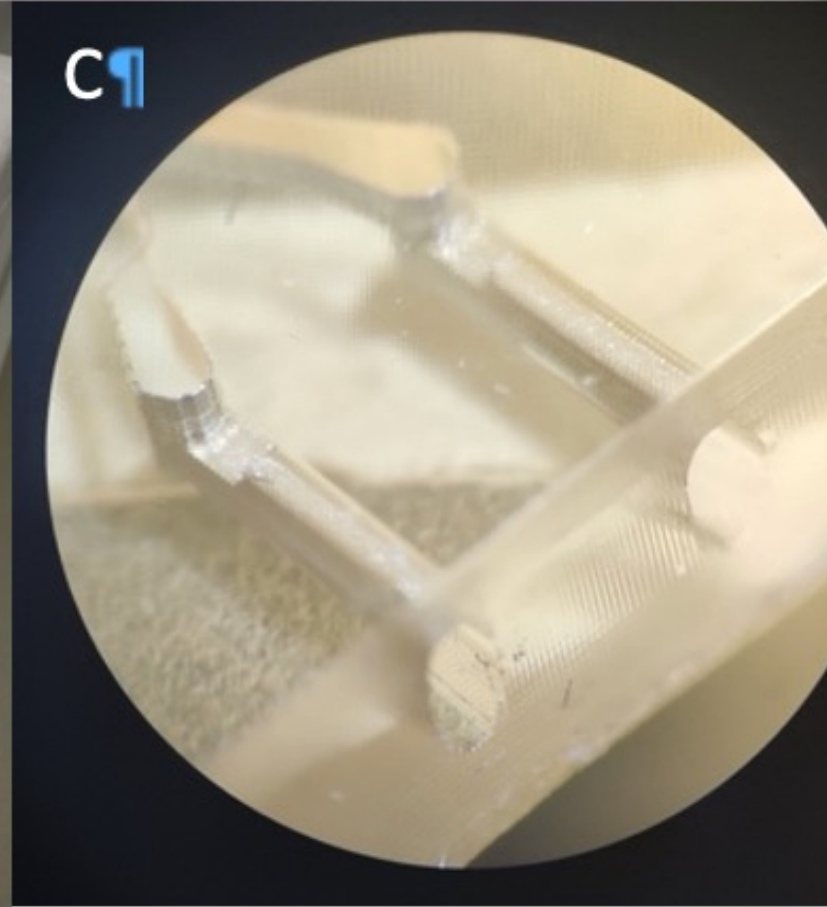

Supplement: Supplementary file 1 [file micromachines-17-00193-s001.zip › Figure S1.pdf]

## Slide 1
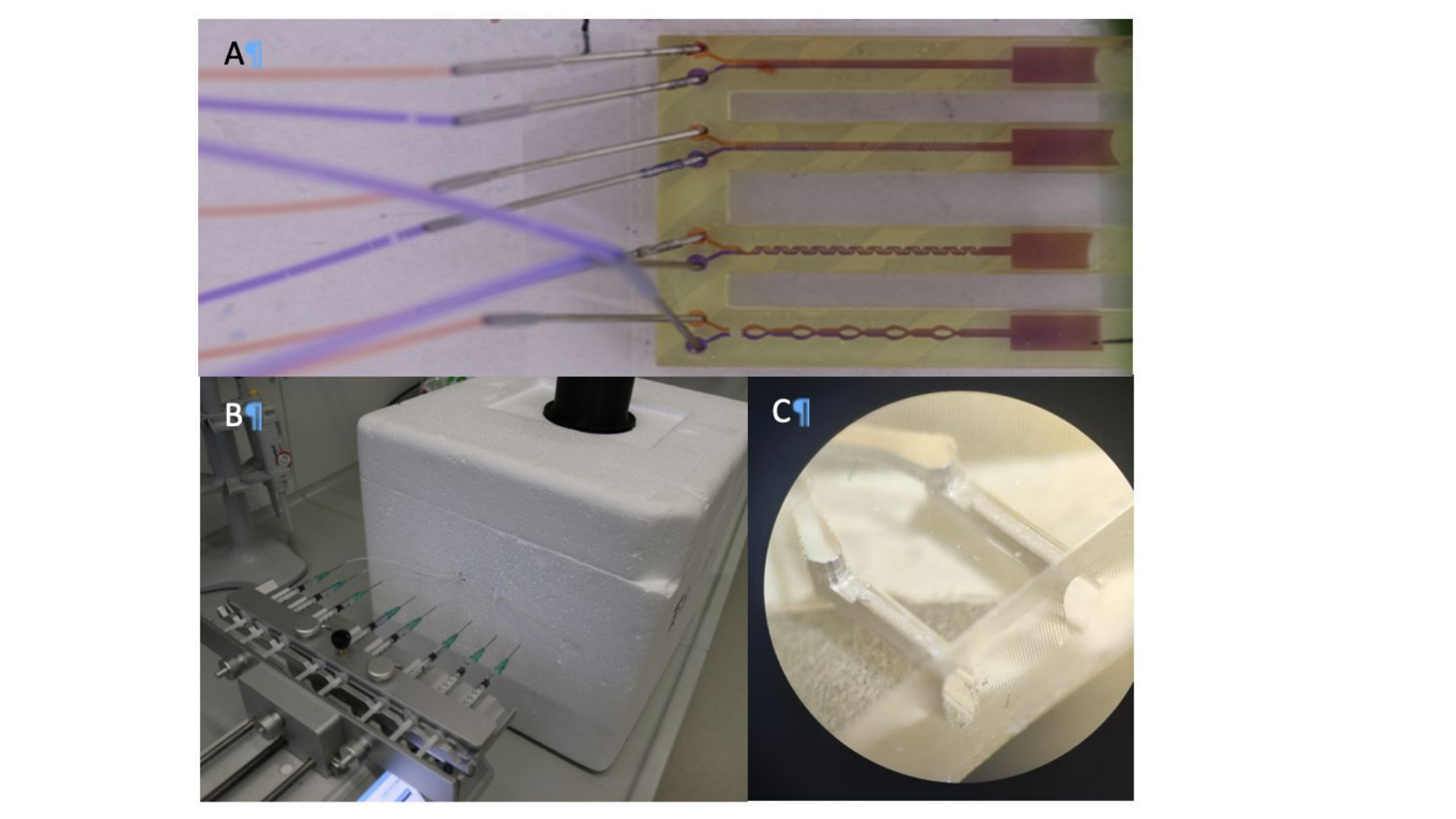

Supplement: Supplementary file 1 [file micromachines-17-00193-s001.zip › Figure S1.pptx]

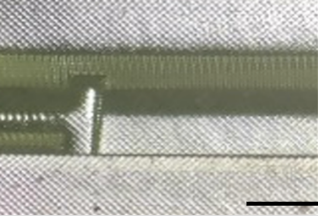

Supplement: Supplementary file 1 [file micromachines-17-00193-s001.zip › Figure S2 Kopie.pdf]

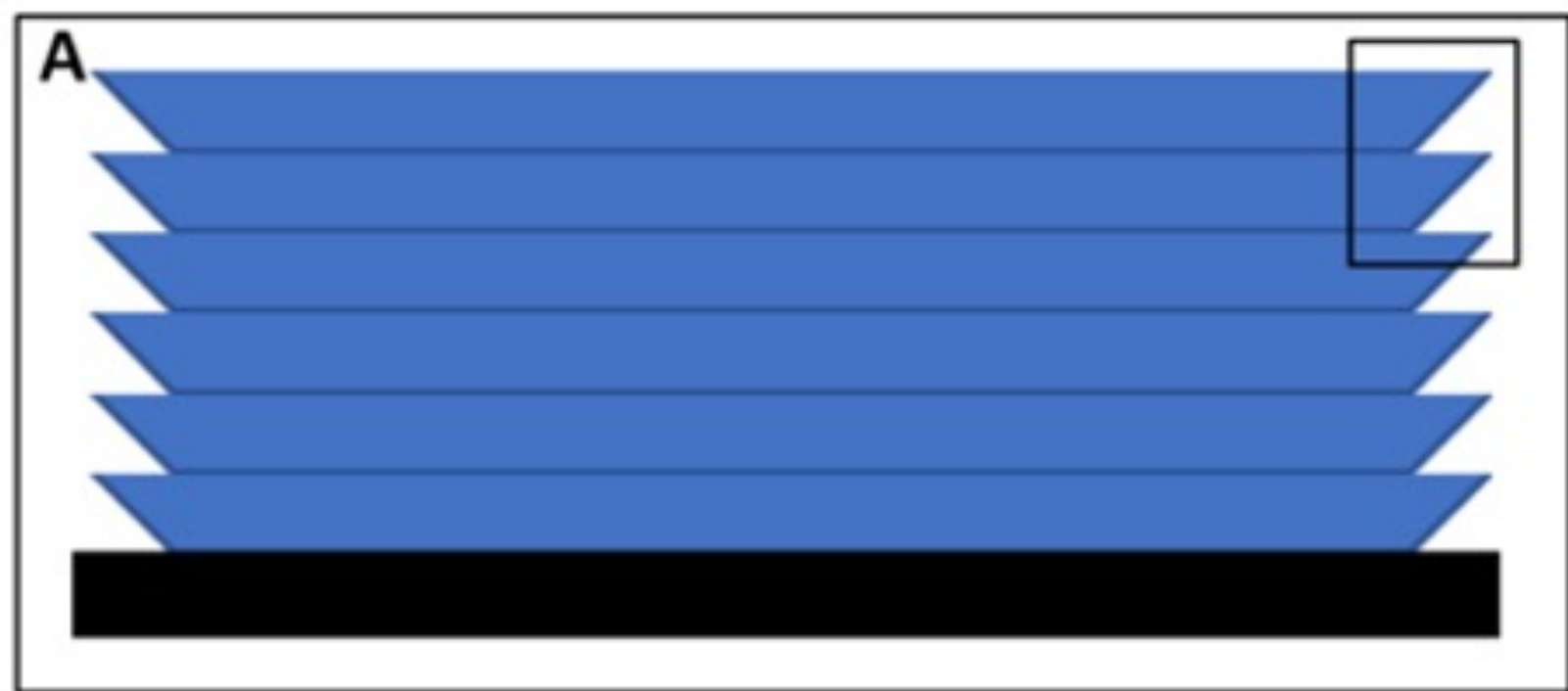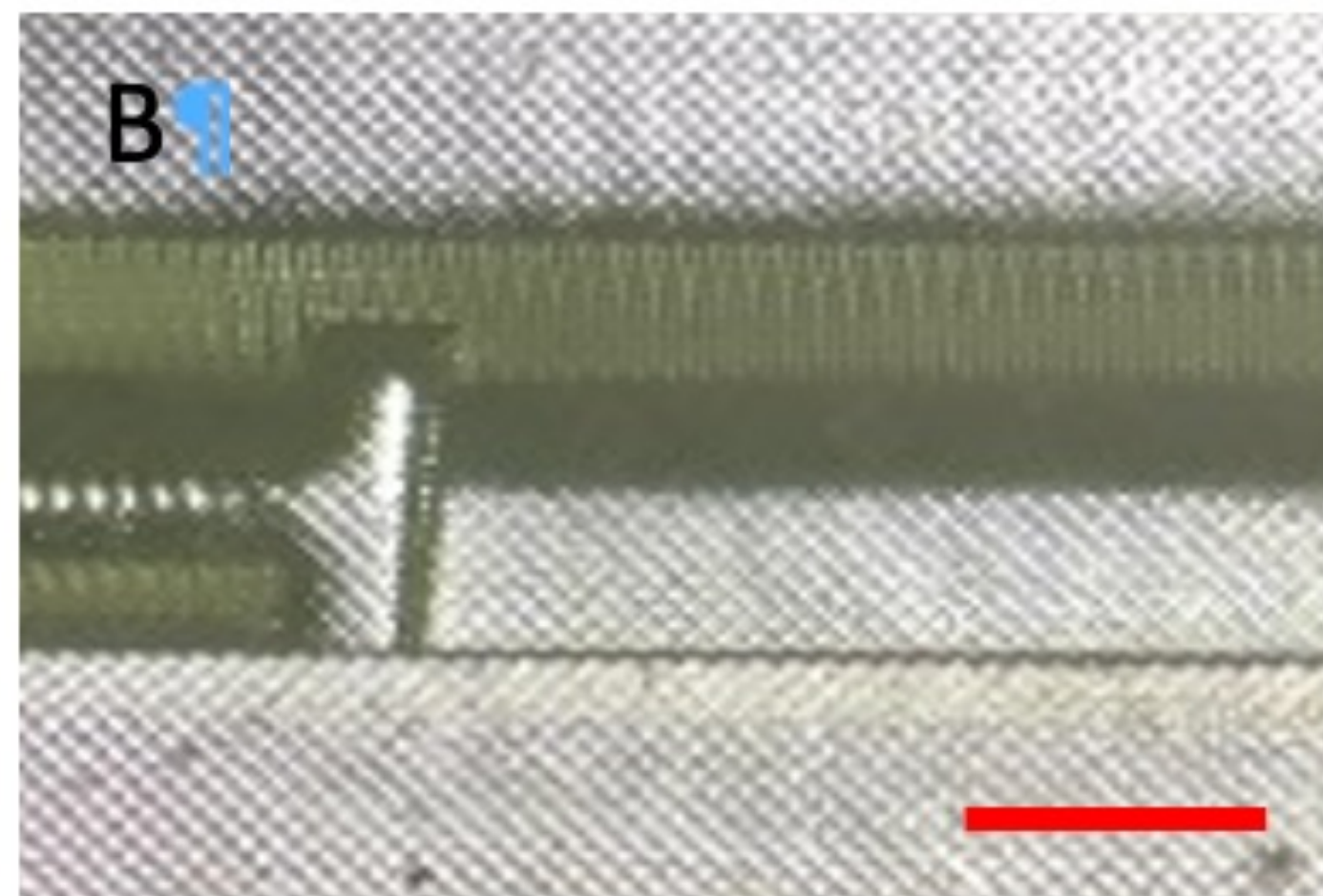

Supplement: Supplementary file 1 [file micromachines-17-00193-s001.zip › Figure S2.pdf]

## Slide 1
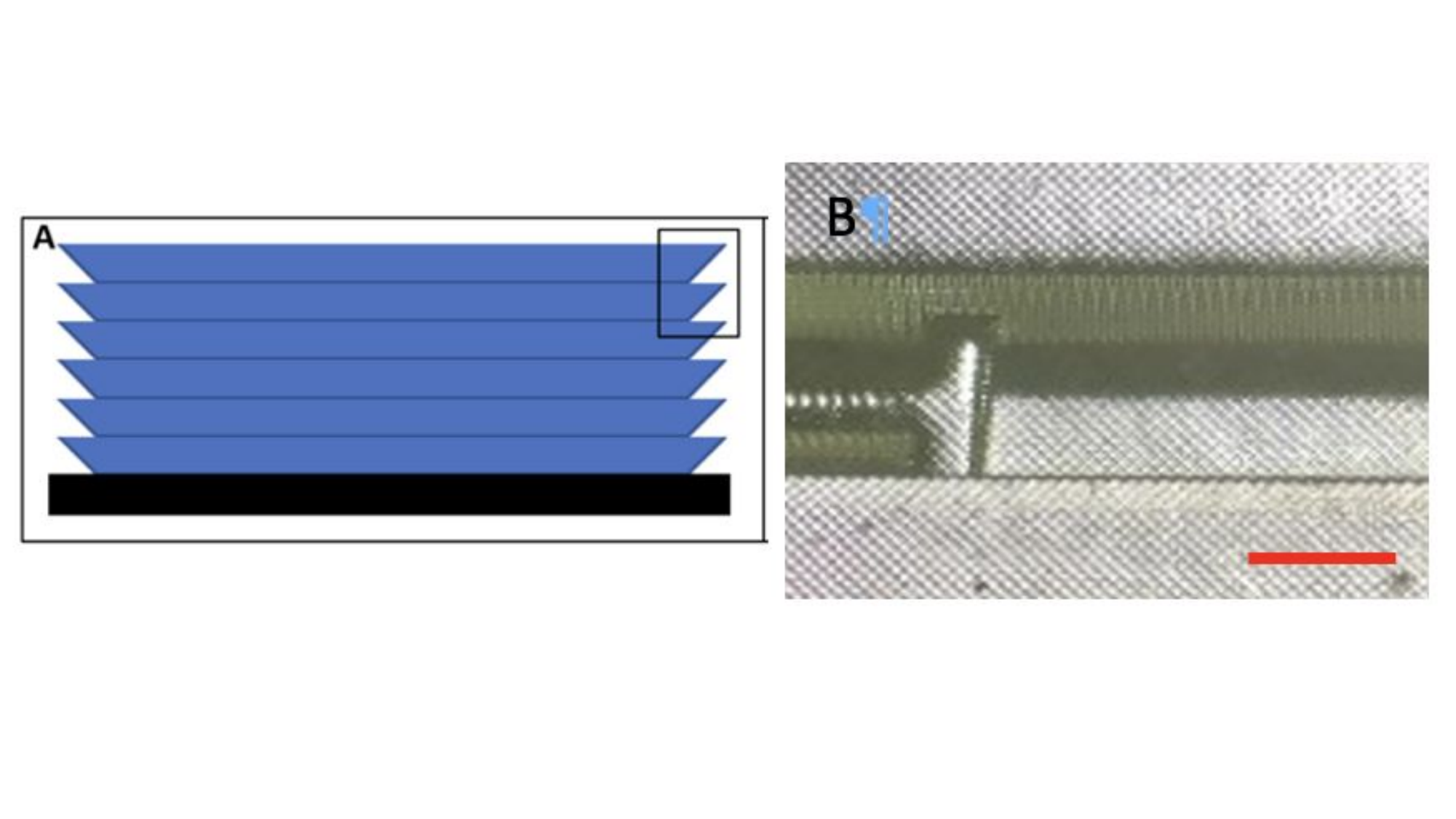

Supplement: Supplementary file 1 [file micromachines-17-00193-s001.zip › Figure S2.pptx]
